# Characterizing Periodic Messaging Interventions Across Health Behaviors and Media: Systematic Review

**DOI:** 10.2196/jmir.2837

**Published:** 2014-03-25

**Authors:** Elaine De Leon, Laura W Fuentes, Joanna E Cohen

**Affiliations:** ^1^Institute for Global Tobacco ControlDepartment of Health, Behavior and SocietyJohns Hopkins Bloomberg School of Public HealthBaltimore, MDUnited States; ^2^Department of Health, Behavior and SocietyJohns Hopkins Bloomberg School of Public HealthBaltimore, MDUnited States

**Keywords:** prompts, periodic messaging, health behavior, review, systematic

## Abstract

**Background:**

Periodic prompts serve as tools for health behavior interventions to encourage and maintain behavior changes. Past literature reviews have examined periodic messages targeting specific behaviors (smoking, physical activity, diet, etc) or media (telephone, email, face-to-face, newsletter, etc) and have found them to be effective in impacting health behavior in the short term.

**Objective:**

Our goal was to review the literature related to periodic messaging and prompts in order to explore typical characteristics, assess the role of prompt timing, identify common theoretical models used, and identify characteristics associated with the effectiveness of periodic prompts.

**Methods:**

Electronic searches of PubMed, PsycINFO, CINAHL, and Web of Science were conducted in October 2012 and May 2013. Database search terms included variant terms for periods, prompts, interventions, media, and health behaviors.

**Results:**

Forty-two of the 55 included research articles found that prompts resulted in significant positive behavioral outcomes for participants. Prompts were delivered via text messages, email, mailed communications, and in a few instances via phone. Generally, the provision of feedback and specific strategies to accomplish behavior change appears to be important for the success of periodic prompts. Rationale for prompt timing was rarely provided, although some studies did organize message content around days of the week or times perceived to be high risk for particular behaviors. Smoking cessation interventions tended to be organized around quit date. Among studies using theoretical models to inform their interventions, the transtheoretical model was most common.

**Conclusions:**

Periodic messaging interventions yield positive results for short-term health behavior changes. Interventions including feedback and prompts that included strategies were more likely to report significantly positive outcomes. Work remains to better understand elements that make periodic prompts successful and whether they are effective in producing long-term outcomes.

## Introduction

Technology has facilitated the delivery of periodic health messages as a low-cost alternative to repeat clinic visits or counseling in an effort to promote health behavior change. Periodic prompts have been delivered through a variety of platforms, including mobile devices. As the use of periodic messages becomes more pervasive in the health intervention literature, it is valuable to examine the characteristics that are associated with the success or failure of periodic prompts.

In 2009, Fry and Neff published a systematic review of periodic prompts in health promotion and health behavior interventions [1]. Fry and Neff focused on literature related to weight loss, physical activity, and diet. They found that periodic messaging had a positive effect, but the effect without personal counseling appeared to wane over time. This result was unclear, however, due to the heterogeneous methods used in the articles reviewed. They also found that when personal feedback was not a part of the intervention, the medium used to communicate prompts did not affect results.

Other literature reviews have also noted the value of periodic messaging. Williams et al, in a review of interventions to increase walking behavior, concluded that non−face-to-face delivery methods may be optimal for walking promotion interventions [2]. Whittaker et al’s systematic review of mobile phone smoking cessation interventions found these interventions are effective at helping people quit smoking in the short term [3]. Cole-Lewis and Kershaw’s review of text-messaging interventions for disease prevention and management found evidence of the short-term effect of these interventions [4]. Text messaging interventions (short message service, SMS) were reported to exhibit good acceptance and short-term efficacy in Wei et al’s review on text messaging for clinical and health behavior interventions [5]. Wei et al agreed with previous reviews that more evidence was needed regarding long-term outcomes.

Recent reviews specific to Web-based interventions have pointed to the importance of better understanding the impact of an intervention’s persuasive features on adherence [6,7]. In a 2012 review, Kelders et al found that significant predictors of improved adherence to Web-based interventions included increased interaction, more frequent updates, dialogue support (including praise, reminders, and suggestions) and participating in the intervention as intended by researchers [7]. Given that prompt characteristics are fundamental elements of periodic messaging interventions, additional investigation is warranted to further identify specific best practices in intervention design.

This review considers literature related to periodic messaging up until May 2013. Whereas previous reviews focused on specific health behaviors or specific media, this review expands upon this body of work by including studies that address a range of health behaviors and employ a variety of media. This review aims to (1) identify prompt characteristics that are associated with intervention effectiveness, highlighting key studies where these elements are closely examined and where lessons can be gleaned, (2) assess whether or not interventions provide rationale for the timing of prompts, and (3) identify common theoretical models used to inform message content.

## Methods

In October 2012, electronic searches were conducted in PubMed, PsycINFO, CINAHL, and Web of Science. This search was not bound by dates. Keywords were customized to optimally search each database and included variant terms for periods (daily, weekly, monthly, periodic, etc), prompts (message, prompt, reminder, notice, etc), intervention (or campaign), medium (telephone, email, face-to-face, newsletter, etc), and health behavior (smoking, drinking, physical activity, diet, etc). Search results were exported into a reference manager where duplicates were eliminated.

Articles that focused on mass media campaigns, websites, knowledge, and/or awareness without addressing a behavioral outcome were excluded. Articles that used periodic messaging as a form of instruction, treatment, and/or therapy (including telehealth) were also excluded as the periodic receipt of the intervention was coincidental and did not provide insight into features used in periodic messaging interventions. Additionally, articles of high intensity interventions perceived to be difficult to replicate on a large scale due to a lack of automaticity in prompting mediums were not considered for inclusion in the final review. Articles were included in this final review if periodic prompts were used as a standalone intervention or as part of a larger program as long as periodic messaging was tied to a primary behavioral outcome, if a biological or behavioral outcome measure was used, and if ongoing health promotion behaviors were targeted. Prompts were considered periodic if they were administered more than twice.

Unique search results underwent a title review to assess relevance. Potentially relevant or ambiguous titles underwent abstract review. Abstracts clearly discussing an intervention including more than two prompts and/or containing specific behavioral outcomes were promoted to “full review”. Abstracts where periodicity was unclear were also promoted at this stage.

Articles that clearly adhered to our inclusion criteria at this stage were included in the final synthesis. In cases where interventions had multiple trials, all trials with different populations were included. In the case of two articles discussing the same trial, the later trial was included. Included articles were assessed using a rating system to quantitatively represent the quality of evidence for each article. Fry and Neff adapted a rating system from a review conducted by Revere and Dunbar [1,8]. Fry and Neff’s [1] adapted rating system is applied here (Table 1).

**Table 1:** Rating system as adapted by Fry and Neff.

Factor	Description	Possible points
Randomization	Assignment to different intervention by chance	2
Control group	Comparison made to group of subjects not given the health behavior intervention	2
**Sampling**		
	Sampling method described	3
	Sample composition clearly described	
	Sample of adequate size	
	Number and ratio of withdrawals described	
**Analysis of main effect variables**		
	Clear definitions for each variable	1
	Clear description of methods and results	
	Numeric table presented for each effect variable	
Follow-up	Follow-up data collection measure effects beyond immediate findings	1
**Content**		
	Intervention clearly described and replicable	1
	Discussion of withdrawals	
	Discussion of study limitations	

## Results

### Summary

**Figure 1:**
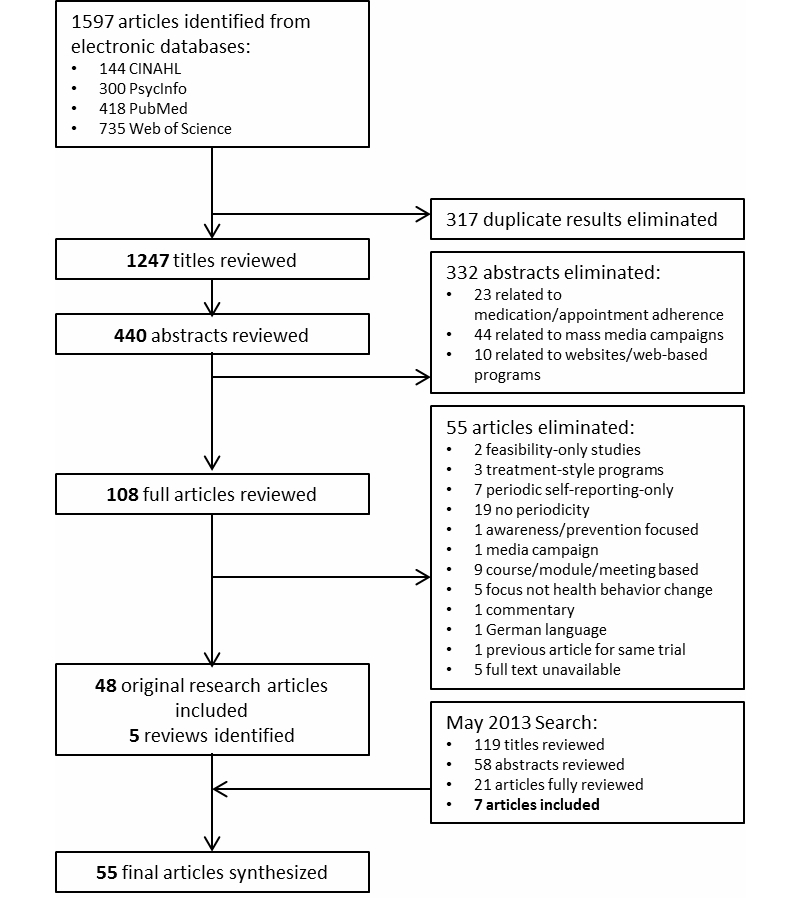
Inclusion and exclusion process.

Searches of the selected databases yielded 1597 search results; of these, 1297 were unique articles. At the conclusion of the review of these titles, 440 were perceived as potentially relevant and underwent abstract review. Of the 440 abstracts reviewed, 108 articles were promoted for full review. Each of the 108 articles was acquired and reviewed with the exception of 5 articles for which full text was unavailable. A final set of 48 articles was included at this stage. In May 2013, an additional 119 titles were identified, 58 abstracts reviewed, and 21 articles were fully reviewed. Of these, 7 articles were included in the final review for a total of 55 articles. An additional 5 reviews on related topics, including Fry and Neff [1], were identified for comparison purposes. Figure 1 depicts the process of inclusion and exclusion for this review.

### Behaviors

A variety of different health behaviors have been targeted using periodic messaging including breast self-examinations [9,10], diabetes management [11-13], diet [14-30], physical activity [15-18,20,21,23-26,28,31-42], smoking cessation [43-56], methamphetamine use [57], sexual health [57,58], mammography adherence [59], and sun protection [60,61] (Multimedia Appendix 1). Of the 55 original research articles using periodic messaging identified by this review, 42 reported significant differences in behavior-related outcomes between intervention and comparison groups across all behaviors, with the exception of sun protection [60,61] and the dietary behavior of iodine consumption [22].

It should be noted that these positive findings only suggest short-term effectiveness due to the limited number of studies with long-term follow-up. Only 5 studies included in this review conducted follow-up beyond 1 month post-intervention [26,29,31,41,56]. Only 12 studies engaged in follow-up beyond the conclusion of their interventions (3 weeks-9 months). Results are mixed for whether these interventions influence long-term behavior change.

### Study Design

Out of 55 studies, 37 included process outcomes as part of their study. A total of 44 studies used a comparison group, where a group either did not receive the intervention or received one of lower intensity, such as a “usual care” intervention, and 30 studies provided participants with feedback regarding their progress.

Multimedia Appendix 2 displays each article’s sample size, health behavior, duration, and design, as well as whether or not studies used controls, performed follow-up, or had additional intervention components. Study design indicates pre-test and post-test when studies involved a statistical comparison between baseline and post-intervention/follow-up measures. Multimedia Appendix 1 presents a condensed version of Multimedia Appendix 2, displaying behavior, purpose of the periodic messaging, tailoring, feedback, medium, and findings.

The purpose of using periodic messages was not consistent across all studies, primarily differing by whether the intervention was intended to deliver educational content, serve as a reminder, or both. In some contexts, prompts were part of a larger intervention, whereas in others prompts were standalone interventions. Of the 13 studies that did not report significant results, 4 provided only health education content via prompts without supplementing this information with strategies and/or counseling. These 4 studies did not provide participants with feedback, such as information about participants’ progress toward health behavior goals, often in relation to milestones set by participants or the intervention. In comparison, of the 42 studies that did report significant results, only 5 delivered education-only prompts without additional tips, strategies, or other forms of guidance, with 2 of these 5 studies including feedback for participants. Thus, the effect of informational content delivery via prompts appears to be enhanced when specific strategies and/or feedback are also provided.

Generally, the provision of feedback appears to be implicated in the success of periodic messaging interventions. Of the 13 studies that did not report significant differences between intervention and comparison groups, 8 did not provide feedback to participants. Conversely, a smaller proportion that did report significant results (17 of 42 studies) did not provide feedback.

### Tailoring

Tailoring prompts was a popular technique used in 34 of the 55 studies. Common tailoring strategies included tailoring using participant name, by baseline characteristics, and for interventions using the transtheoretical model, by stage of change. Only a few studies were designed to assess the effect of tailoring, comparing tailored to untailored prompts [14,17,19,36]. Hageman, Noble, and Pullen were unable to identify a significant difference in physical activity for older women who received tailored newsletters versus those who received a standard newsletter. Heimendinger et al did not find any enhanced effect on fruit and vegetable consumption by tailoring materials when participants received only one mailing, but it was advantageous when participants received four mailings, with tailored materials “more likely to be remembered, read, and appreciated” [19]. Like Heimendinger et al, Allicock et al found that the more content read from tailored newsletters and the more personally applicable readers felt the information was, the more they increased fruit and vegetable consumption [14]. De Vries et al also identified that participants receiving tailored mailings read more of the information provided than participants who received generic letters [17].

### Medium

Prompts were delivered via different media across interventions, with the majority delivered via mobile messaging [11,12,20-24,35,37,42,43,45-47,49,50,52-58,62], followed by print communications [9,10,14,17,19,20,24,25,27,29,30, 32,33,36,39,40,42,48,59,63], email [15,18,26,28,31,34,38, 44-47,51,58,60], telephone [10,13,14,24,32, 33,39,41,45,46], and even newspaper articles [16]. The medium did not appear to impact the success of an intervention in eliciting behavior change.

### Frequency

Frequencies for prompts included multiple prompts per day, daily, several times a week, weekly, monthly, bi-monthly, and even yearly (Multimedia Appendix 2). Some interventions varied their periodicity depending on progress through the intervention. This pattern was particularly dominant in smoking cessation interventions that often used quit date or stage of change to moderate the intensity of prompts [43-47,49,51-53,55,56,62]. Frequency of messages tended to be of higher intensity before and immediately following the quit date.

Interventions with both very high frequency of prompts as well as interventions of low or irregular frequency yielded positive results. With regard to low frequency, 3 studies with a monthly frequency did not report significant results, none of which provided feedback. Ten studies of monthly frequency did report significant results, 4 of which provided participant feedback. The vast majority of studies using monthly or less frequent prompts were print-based interventions.

Of the 55 studies included in this review, only Haug et al considered variation in frequency by examining whether or not frequency of text messages (1 versus 3 weekly) could impact smoking cessation [50]. No difference was identified between these two conditions.

### Timing

None of the studies included in this review provided an explicit rationale for the pattern of messaging in their interventions. In most of the few studies that described the pattern and/or timing of messaging, the decision-making process of why a particular approach was taken was not discussed. Studies that described their pattern for prompt delivery are discussed further here.

Patrick et al’s text messaging intervention for improved diet and increased physical activity [24] identified through their formative research that preferred number of messages per day varied across participants. During implementation, researchers chose to provide flexibility in the number and daily timing of messages. If a user did not respond to a message, the number of messages requesting replies was reduced in an effort to minimize annoyance. Overall, the intervention consisted of weekly topics, where messages related to the topic were sent on Mondays, Wednesdays, and Saturdays. Tips or questions were sent on Tuesdays, Fridays, and Sundays. The latter messages were tailored by eating behaviors.

Arora et al similarly used days of the week to dictate the information sent via text messages in a proof-of-concept intervention for inner city patients with poorly controlled diabetes [11]. Messages were sent three times daily, and prompts were organized by time of day as well. Prompts sent at 9 a.m. took several forms: (1) medication reminders were sent Mondays and Wednesdays, (2) healthy living challenges were sent Tuesdays and Saturdays, (3) trivia messages were sent Thursdays, and (4) a phone number for free diabetes management gifts were sent on Sundays. At all other times (noon and 6 p.m. daily), messages sent were meant to educate/motivate participants.

In an intervention targeting waist circumference and blood pressure, Park and Kim sent prompts via text messages three times weekly [23]. Monday messages were meant to “greet the new week”; messages delivered Wednesdays encouraged lifestyle modification. The third message of the week was sent on Fridays, and researchers used participant self-monitoring data to offer personalized recommendations for diet and exercise. For Reback et al’s intervention to reduce methamphetamine use and high-risk sexual behaviors among men who have sex with men, study staff responded to participant text messages during hours that were identified in a formative stage as time when high-risk activity would occur [57]. It was unclear whether or not prompts were also sent during these high-risk times.

Other interventions chose to send prompts at the end of the week in order to counter potentially heightened risk during the weekend. Lim et al’s sexual health intervention sent text messages to participants every 3-4 weeks, and although messages were sent at “various times and on different days”, the majority of their text messages were sent on Friday and Saturday evenings while monthly emails about different safe sex or sexually transmitted infection (STI) topics were sent during the work week [58]. Dixon et al’s sun protection intervention emailed UV forecasts to participants at the end of the working week to prompt sun protection behaviors over the weekend [60].

In Gierisch et al’s mammography adherence intervention, reminders were delivered 2-3 months prior to participants’ mammography due dates [59].

### Theoretical Underpinnings

Kim and Glanz argued that health communications interventions are most effective when grounded in behavioral theory [37]. Several studies utilized theoretical models for behavior change to inform the content of prompts and/or to tailor prompts for individuals. The transtheoretical model (TTM), was the most popular foundation for the interventions identified [17,19,26,30,32-34,39,41,48,50,51,53]. Other theoretical models employed include social cognitive theory (SCT) [17,19,21,26,27,29,32,33,39,57], the theory of planned behavior (TPB) [17,26,33,48,59], the health belief model (HBM) [17,19,57,59], self-determination theory (SDT) [27], precaution adoption model (PAM) [17,30], goal setting theories [13,15,17,19,21,30,32,34,35,39,43,59], the elaboration likelihood model [59], and behavioral self-regulation [52,53]. Some studies chose several different models and/or theories to draw upon. For example, de Vries developed the I-change model, which integrates TPB, SCT, HBM, TTM, PAM, and goal-setting [17].

Two interventions directly compared theoretical models to inform the tailoring of prompts. Resnicow et al compared SCT to SDT, which “differentiates between autonomous and controlled behavioral regulation” as the building blocks for tailoring newsletters designed to increase fruit and vegetable consumption [27]. Participants who received newsletters tailored using the SDT and motivational interviewing principles/strategies perceived their newsletters as marginally more relevant than participants who were provided SCT-based newsletters. However, no between-group differences in fruit and vegetable intake at 3 months were found; each group showed similar improvement.

With regard to support theories, Gabriele et al tested the effects of nondirective and directive e-coach support on weight loss [18]. Researchers hypothesized that individuals in the nondirective support condition (“cooperation and accepting the support recipient’s thoughts and choices”) would show greater changes in diet and physical activity than those in the directive support condition (“prescriptive and guided by rules”). However, Gabriele et al found greatest loss of weight and waist circumference for directive support participants.

### Participant Feedback

Information regarding participant experience was solicited in 28 of the included 55 studies. Multimedia Appendix 2 summarizes information regarding participant feedback for all studies where this information was available. Overall, participants expressed that prompts were helpful and reported high satisfaction with the interventions for which this information was available [11,14,15,20,24,36,39,44,45, 48,52-54,58]. Participants reported reading emails with less frequency than text messages [43]. In another intervention, email was preferred over paper or website [26]. Tailored material was perceived by participants to be personally meaningful and/or helpful [14,15,17,19,42,63]. Participants in Faridi et al’s text messaging diabetes management intervention reported that low adherence was likely due to an interface that was not user-friendly or was due to inexperience with mobile phone use [12]. Fjeldsoe et al suggested that assessment burden was a possible reason for attrition in their text messaging exercise intervention [35].

## Discussion

### Principal Findings

Periodic prompts have been used extensively in health behavior interventions over the past decade. This review identified 55 original research articles related to a variety of behaviors, with 42 bearing evidence of short-term behavioral changes, and 3 studies additionally suggesting long-term behavioral changes. Given that the included interventions varied by many factors, including behavior, prompt, use of feedback, goal-setting, and theoretical models, it is difficult to form a conclusive judgment regarding which combination of elements is most effective. What is clear, however, is that periodic messaging has positive short-term effects across a number of health behaviors and across media and frequency. Unfortunately, the sustainability of these outcomes remains a concern due to the small number of studies that conducted follow-up beyond the conclusion of their interventions.

Only a small subset of studies compared the use of specific features, thus little is known regarding best practices to appropriately leverage tailoring, frequency, timing, and the use of theoretical models in periodic prompting interventions. For those looking to structure periodic messaging interventions, participants tend to read more of the information provided in tailored materials versus untailored materials [17,19]. It also appears that the effect of tailoring is more evident upon the receipt of more than one tailored message [14,19]. Generally, participants show preference for tailored messages/materials over untailored content [14,15,17,19,42,63]. Directive support appeared to be more beneficial in impacting weight and waist circumference than nondirective support [18]. Participants appear to prefer text messages over emails [43] and emails over paper materials or websites [26]. Additionally, this review has identified feedback as an important factor in determining prompt intervention success. Studies with significant results tended to provide participants with feedback regarding progress in making a given health behavior change. Interventions with prompts delivered on a monthly basis did better if they included feedback. Enhancing prompt content with specific strategies also appeared to be associated with intervention success.

All media, including printed/mailed materials, were successful in encouraging health behavior changes, though text messaging allowed for the greatest number of prompts to be delivered on a daily basis. Unfortunately, information regarding whether particular patterns of messaging are most effective for specific behaviors and media is unclear. Authors of the included trials offer sparse rationale for decisions regarding the timing and frequency of their interventions. Only Haug et al compared prompt frequency (1 vs 3 text messages weekly) and found no differences between the two frequency conditions [50]. This missing information would be incredibly valuable as prompt frequency and days of prompt delivery may be most impactful for participants. For smoking cessation, periodic messaging interventions tend to use quit day to dictate the crescendo of prompt frequency, and still other interventions targeted times when they felt participants would be most at risk for engaging in negative behaviors. Other interventions may also benefit from targeting specific days and/or time of the week where prompts would be most likely to be acknowledged.

Although we characterize the use of comparison groups, process evaluation, follow-up, feedback, tailoring, and other characteristics of these interventions, the espousing of specific facets as “best practice” is a challenge and a limitation of this review. Typical attrition for these interventions was also difficult to assess due to the automated nature of many of these prompts (ie, participants may not have formally withdrawn from the study but may stop acknowledging the majority of messages sent). As is the nature of all reviews, the reach of articles reviewed is another limitation, reflecting the search terms used in the databases utilized. It is also possible that our inclusion/exclusion criteria may have excluded articles that could have also characterized the nature of periodic messaging.

This review sought to identify the behaviors that have been targeted by periodic prompts, the outcomes of these interventions, and the variety of approaches that have been used, in order better understand how to best use periodic prompts for health behavior change. Our review is robust due to our systematic approach; we were able to ensure that we were inclusive with regard to both prompt medium and targeted health behaviors. Additionally, each study was rated in such a way that allows for between-study comparisons despite the diversity in study design.

### Conclusions

In light of this review, it is suggested that future periodic messaging interventions be conducted purposefully in order to better examine what elements make these interventions successful. Although this review identified cases that compared frequency of contact, prompt medium, and theoretical frameworks, work remains to be done to identify best practices for these interventions. Additionally, future studies should communicate the rationale behind decisions regarding the pattern and content of messages to identify whether or not there are optimal times and situations when these messages should be administered for particular populations. Finally, future periodic messaging interventions should consider the long-term benefits of the intervention by observing a substantial follow-up period post intervention. Despite the breadth of literature demonstrating the use of periodic messaging for several health behaviors, much work remains to be completed that provides evidence for the frequency and timing of the messages and sustainability of outcomes for these interventions.

## Multimedia Appendix 1

Summary table of purpose, features, and medium for included periodic messaging interventions.

## Multimedia Appendix 2

Features of included periodic messaging health interventions.

## Abbreviations

HBM: health belief model
PAM: precaution adoption model
SCT: social cognitive theory
SDT: self-determination theory
SMS: short message service
STI: sexually transmitted infection
TPB: theory of planned behavior
TTM: transtheoretical model
