# Supplementary material for: Characterizing Periodic Messaging Interventions Across Health Behaviors and Media: Systematic Review
Source: J Med Internet Res. 2014 Mar 25;16(3):e93. doi: 10.2196/jmir.2837 (PMC3978550; doi:10.2196/jmir.2837)
Supplement: Supplementary file 1 [file jmir_v16i3e93_app1.pdf]

Multimedia Appendix 1. Summary table of purpose, features, and medium for included periodic messaging interventions.

Author	Behavior	Purpose of the Periodic Messaging	Tailoring	Feed-back	Medium	Findings
eHealth						
Abroms 2012 [43]	smoking	content delivery and encouragement	yes, by participant name, quit date, gender, reasons for quitting, money saved, social support, smoking triggers, and use of smoking medication	yes	text messages	all participants made one quit attempt post-enrollment; only 14.3% reported not smoking at 4 week follow up
Abroms, 2008 [44]	smoking	counseling follow-up after 15 min counseling session	yes, by participant's reasons for smoking, triggers, quit data, and other baseline information	no	email	intervention group participants more likely to report quit attempt and to have used program recommended strategies to cope with cravings
Aitsallo 2012 [31]	PA	content delivery and encouragement of tailored step goals	yes, step goals tailored based on individual's baseline activity	no	email	at 6 and 12 months follow-up, the intervention achieved only modest differences between intervention and control groups in using stairs and walking for leisure
Arora 2012 [11]	diabetes manageme nt	content delivery, medication reminders, encouragement	no	no	text messages (English and Spanish)	participants reported increased fruit and vegetable intake, exercise, and foot checks compared to before intervention; 70% of participants completed at least

Block 2008 [15]	diet or PA	content-delivery, weekly goals	yes, by chosen behavior (PA or diet), children in the home, cooking habits, PA location preferences, specific foods, goals perceived as achievable	yes	email	4 out of 6 healthy challenges significantly greater likelihood of improved self-assessed health status in the intervention group; intervention group also saw improvements in self-efficacy towards changing diet
Borland 2012 [47]	smoking	content delivery, advice, encouragement	yes, by stage/perspective on change, quit date, time quit, years smoked, have children, live with non-smokers, sex, age	yes	email and/or text messaging	study failed to find clear significant effects between the intervention conditions and the control, possibly due to low rates of intervention uptake, contaminating effects of use of alternative interventions by controls, and inadequate power
Brendryen 2008a [46]	smoking	content delivery, awareness, encouragement	no	yes	day specific webpages, email, text messages, interactive voice response	abstinence rates significantly higher for intervention group than control at 1, 3, 6, and 12 months (ratio of abstinence similar at each time point)
Brendryen 2008b [45]	smoking	content delivery, awareness, encouragement	no	yes	day specific webpages, email, text messages, interactive voice response	abstinence rates significantly higher for intervention group than control at 1, 3, 6 months, and marginally significant at 12 months; lack of significance due to subjects in the control condition performing a second quit attempt, not due to intervention group relapse
Dinger 2005 [34]	PA	content delivery	no	no	email	preliminary evidence showed

		(strategies)				that participants significantly increased walking minutes post-intervention (median 55 min/wk at baseline to median of 245 min/wk post-intervention)
Dixon 2007 [60]	sun protection	delivery of UV forecasts, protective recommendations	yes, by first name	no	email	sun protection behaviors and reported sunburn did not differ for participants receiving UV forecasts (with or without behavioral prompts) compared to those receiving standard forecasts
Faridi 2008 [12]	diabetes manageme nt	feedback and reminders	yes, by feedback from glucometer and pedometer	yes	text messages	intervention did not affect any clinical outcomes to a statistically significant level; produced significant changes in self-efficacy
Fjeldsoe 2010 [35]	PA	content delivery (strategies), goal checking	yes, by first name	yes	text messages	participants in intervention group increased frequency of PA and walking for exercise frequency at 13 weeks post-intervention
Free 2011 [49]	smoking	content delivery (strategies), encouragement	yes, by demographic and other information provided at baseline	yes	text messages	biochemically verified continuous abstinence significantly increased in intervention group
Gabriele 2011 [18]	diet and PA	feedback	yes, by support type	yes	email	participants in the directive supportive condition experienced a greater decrease in weight and waist

						circumference than nondirective or minimal support conditions; participants across conditions lost an average of 8.53 pounds
Hageman 2005 [36]	PA	content delivery	yes, by responses to baseline assessment (self-reported PA, benefits and barriers to activity, and self-efficacy and initial goals for activity)	no	newsletter	self-reported PA did not increase for either groups though specific biomarkers did improve (increased flexibility for both groups, maximal oxygen consumption and percent body fat decreased in standard group, but no effect with intervention group);
Haug 2009 [50]	smoking	stage of change assessment, feedback	yes, by baseline assessment and stage of change	yes	text messages	preliminary results show no difference in groups in cigarettes smoke, quit attempts, stage of change, self-efficacy, or decisional balance
Joo 2007 [20]	diet and PA	content delivery, recommendations	yes, individually prescribed amounts and types of food	no	text messages, information brochures	over two-thirds of subjects experienced a reduction in waist circumference of 5-7.5 centimeters
Kim 2013 [37]	PA	encouragement	no	no	text messages	participants in the intervention significantly increased their number of steps
Lenert 2004 [51]	smoking	"individually timed educational messages," content delivery	yes, by stage of quit effort	yes	email	enhancing a smoking cessation website with email prompts at strategic times increased rate of early successful quit efforts

Liguori 2007 [38]	PA	content delivery	no	no	email	no significant difference from initial activity levels within intervention or control; participants that were assumed to have read emails did have significant higher PA scores when compared to control
Lim 2012 [58]	sexual health	content delivery	no	no	text messages and email	no significant difference between groups in reported condom use with risky partners; female participants in the intervention group more likely to have STI test compare to control group female subjects
Lombard 2010 [21]	diet and PA	content delivery follow-up after 4 in-person class sessions (1 hour)	no	no	text messages	intervention group lost weight while the control group gained weight; difference observed between intervention and control groups for total cholesterol, self-management, and PA scores
Mehran 2012 [22]	diet	content delivery	no	no	text messages	no significant difference observed in iodine-related behaviors between the intervention group and control group; differences seen in knowledge and attitudes.
Obermayer 2004 [52]	smoking	content delivery (strategies), reminders	yes, by probable high-risk situations	yes	text messages	43% of participants had made at least one 24 hour quit

						attempt; 10 of 27 had quit at posttest and 15 participants reduced consumption from average of 76 cigarettes to 32 cigarettes per week
Park 2012 [23]	diet, PA, and blood pressure	content delivery (strategies), reminders, feedback	yes, by self-monitoring data	yes	text messages	intervention group experienced decreased waist circumference, body weight, systolic and diastolic blood pressure, and total cholesterol whereas the control group saw increases in all of these measures; no difference observed in fasting blood glucose
Patrick 2009 [24]	diet and PA	content delivery, feedback	yes, by number and timing of receipt of messages, participant's eating behaviors and previous replies	yes	text messages (SMS and MMS), printed materials, phone	intervention group lost more weight than the usual care group; weight loss for the intervention group was modest
Plotnikoff 2010 [26]	diet and PA	content delivery	no	yes	email	positive effects observed following initial intervention maintained or were additionally increased 6 months later; a similar impact on behavior was seen for the control group from receiving the health promotion messages in one bulk message
Reback 2012 [57]	methamphe tamine use	"risk reduction messages"	yes, by responses to five brief questions regarding	yes	text messages	at 2 month follow-up, participants had significant

	and sexual health		methamphetamine use			decreases in frequency of methamphetamine use and unprotected sex while under the influence and reductions of unprotected anal intercourse with HIV-positive partners
Riley 2008 [53]	smoking	content delivery, encouragement to remain smoke free during high risk times	yes, by stage of change and by probable high-risk situations	no	text messages	45% of participants report 7 day point prevalence abstinence (42% biochemically verified)
Rodgers 2005 [62]	smoking	content delivery (strategies), encouragement	yes, by participant characteristics and nickname	yes	text messages	intervention group had twice as much quitting than control group at six weeks; between group difference unclear at six months but quitting remained high for intervention group
Shapiro 2012 [42]	diet and PA	content delivery, feedback	yes, by number and timing of receipt of messages, participant's eating behaviors and previous replies	yes	text messages (SMS and MMS), printed materials	weight lost modest; number of steps increased for the first 6mos, leveling off thereafter
Snuggs 2012 [54]	smoking	motivational support, encouragement of medication use	yes, by participant name and medication choice	yes	text messages	At six months, 38% of the total number of participants reported 7-day point prevalence abstinence; 23% of the total was CO-validated
Thomas 2011 [28]	diet and PA	content delivery (strategies)	no	no	email	intervention group maintained weight loss which was significantly greater than that maintained in the control group; control group exhibited

						more rapid decline in weight loss maintenance
Whittaker 2011 [55]	smoking	content delivery, role modeling	yes, participants selected role models from whom they received messages	no	video messages (phone-delivered)	no significant differences in 7-day point prevalence abstinence between intervention and control groups at 4 or 12 weeks, or 6 months (study underpowered); majority of participants in both groups made multiple quit attempts
Ybarra 2012 [56]	smoking	content delivery, encouragement, recommendations	yes, by participant stage in quitting process	yes	text messages	cessation at 3 months did not differ significantly from intervention group
Non-eHealth						
Albright 2005 [32]	PA	reinforcement and reminder of content of primary intervention	yes, participants in phone condition received personalized counseling	yes	newsletter/phone + newsletter	both groups initially increased PA; after 10 months, phone and mail group sustained significantly increased PA while mail-only group relapsed to baseline PA
Allicock 2010 [14]	diet	content delivery	yes, by use of baseline questionnaire responses TCs were tailored	yes	tailored newsletter, phone calls	increased exposure to TCs and increased perceived value of TCs resulted in increased FVC; MI calls not associated with FVC
Carter-Edwards 2009 [16]	diet and PA	content delivery, advice	no	no	newspaper articles (print and Web site)	participants throughout the duration of the study lost an average of 6 lbs. (3.2% of initial weight)
Conn 2003 [33]	PA	second intervention	no	no	phone calls,	participants receiving prompts

		after small group individual sessions, content delivery and encouragement			newsletter, pamphlet, and recipe card	performed significantly more exercise than those who did not
Crane 2006 [61]	sun protection	recommendations for child sun care	no	no	sun protection packets	small but significant differences in overall use of sun protection strategies; small impact on tanning and freckling
de Vries 2008 [17]	diet, PA and/or smoking	feedback, action plans	yes, by behavior, responses to three questionnaires and person's stage of change	yes	letters	for diet and physical activities, participants receiving tailored information experienced significantly more improvement compared to the control group; no differences between groups existed for smoking due to high cessation in both groups
Etter 2001 [48]	smoking	personalized counseling, content delivery	yes, by participant responses to questionnaire and stage of change	no	counseling letters and two stage-matched booklets	abstinence 1 month post-intervention was 2.6 times greater in the intervention group than in control group (an additional quitter for every 28 participants)
Gierisch 2010 [59]	mammogra phy adherence	reminders	yes, supplemental interventions (priming letter and call) were tailored by women's perceived barriers to mammograms	no	printed letter reminders or printed booklets or telephone	all reminder types equally effective (averages of 50-60 days/year non-adherent), automated telephone reminders least expensive
Grady 1984 [9]	breast self-	"cue enhancement,"	no	no	postcard	for both cyclic and noncyclic

	exam	reminders				women, self-management and postcards led to highest BSE rates; for cyclic women who only received postcards, there was no increase in BSE; for noncyclic women rates of BSE in combined condition match postcard only
Heimendinger 2005 [19]	diet	content delivery, skill-building	yes, by responses to baseline questionnaire (ST, MT, MRT) and telephone follow-up interviews at 5 months (MRT)	yes	print communications (booklet, newsletters, final letter)	linear trend and increased consumption of 0.21 servings across experimental conditions of increasing intensity (SU<ST<MT<MRT)
Marcus 2007 [39]	PA	feedback	yes, by feedback generated by computer expert system	yes	phone, printed reports	both intervention groups reported increase in PA from baseline to 6 months; a significant difference in increased PA only evident in print group from baseline to 12 months
Mayer 1986 [10]	breast self-exam	reminders after a 1hr teaching workshop	no	yes	phone or postcards	participants receiving phone prompts more likely to practice BSE at least once compared to mail prompts; both phone and mail prompt condition were more likely to perform BSE once as compared to the control group
Opdenacker 2008 [40]	PA	reminder follow-up from group meeting,	no	no	letters	no significant difference at pretest or posttest between

		content delivery (strategies), encouragement				intervention or control for self-reported outcome measures; activity counts increased in the intervention group as measured by accelerometer (not confirmed by self-report)
Phelan 2011 [25]	diet and PA	reminders, feedback	no	yes	postcards	normal weight women in the intervention group less likely than those in standard care group to gain above recommended; no difference seen between groups for overweight/obese women
Pinto 2006 [41]	PA	counseling, PA monitoring, health problem identification	no	yes	phone, exercise tip sheet	intervention group reported significantly more minutes of PA, moderate-intensity PA, and higher energy expended as compared to the contact control group; no differences in BMI or percent body fat
Resnicow 2008 [27]	diet	content delivery	yes	no	newsletter	no between group differences in fruit and vegetable intake at 3 months though each group showed similar significant increase in FVC (one for short questionnaire, half for long questionnaire)
Resnicow 2009 [63]	diet	content delivery	yes, by participant newsletter preference and graphics	no	newsletter	no between group differences in FVC at 3 month; each group showed similar significant increase in intake

Sacco 2004 [13]	diabetes manageme nt	content delivery, emotional support, counseling, monitoring	yes, by participant preference, out-of-range glucose values, and new goals	yes	phone	coaching group's mean HbA1c was significantly lower while the control group's was significantly higher
Williams-Piehota 2004 [29]	diet	content delivery	no	no	pamphlets and other promotional items	all participants increased number of fruits and vegetables consumed (2.69 to 3.86) and maintained increase to 4 month follow-up; no differences by message-type
Wright 2011 [30]	diet	feedback	yes, by stage and processes of change constructs and responses to food frequency questionnaire	yes	mailed printed dietary feedback reports	participants receiving printed feedback were more effective in increasing fruit intake than small group nutrition education sessions and equally effective in improving saturated fat intake; no dietary improvements seen in cereals and grains for any group of participants
